# Engaging cervical spinal circuitry with non-invasive spinal stimulation and buspirone to restore hand function in chronic motor complete patients

**DOI:** 10.1038/s41598-018-33123-5

**Published:** 2018-10-19

**Authors:** Yevgeniy Freyvert, Nicholas Au Yong, Erika Morikawa, Sharon Zdunowski, Melanie E. Sarino, Yury Gerasimenko, V. Reggie Edgerton, Daniel C. Lu

**Affiliations:** 1 Department of Neurosurgery, University of California, Los Angeles, Los Angeles, California, 90095 USA; 2 Neuromotor Recovery and Rehabilitation Center, David Geffen School of Medicine, University of California, Los Angeles, Los Angeles, California, 90095 USA; 3 Departments of Integrative Biology and Physiology, University of California, Los Angeles, Los Angeles, California, 90095 USA; 4 Neurobiology, University of California, Los Angeles, Los Angeles, California, 90095 USA; 5 Brain Research Institute, University of California, Los Angeles, Los Angeles, California, 90095 USA; 6 Rancho Los Amigos National Rehabilitation Center, Downey, California, 90242 USA; 7 Pavlov Institute of Physiology, St. Petersburg, Russia

## Abstract

The combined effects of cervical electrical stimulation alone or in combination with the monoaminergic agonist buspirone on upper limb motor function were determined in six subjects with motor complete (AIS B) injury at C5 or above and more than one year from time of injury. Voluntary upper limb function was evaluated through measures of controlled hand contraction, handgrip force production, dexterity measures, and validated clinical assessment batteries. Repeated measure analysis of variance was used to evaluate functional metrics, EMG amplitude, and changes in mean grip strength. In aggregate, mean hand strength increased by greater than 300% with transcutaneous electrical stimulation and buspirone while a corresponding clinically significant improvement was observed in upper extremity motor scores and the action research arm test. Some functional improvements persisted for an extended period after the study interventions were discontinued. We demonstrate that, with these novel interventions, cervical spinal circuitry can be neuromodulated to improve volitional control of hand function in tetraplegic subjects. The potential impact of these findings on individuals with upper limb paralysis could be dramatic functionally, psychologically, and economically.

## Introduction

Over 2.5 million people are afflicted with chronic spinal cord injury (SCI) with over 130,000 newly afflicted individuals each year worldwide^1^. The societal and personal impact of SCI are significant, due in large part to the peak incidence age of 30 or younger^2^. Surveys of tetraplegic SCI subjects have revealed that regaining upper extremity and hand function ranked most important^3^, as even partial restoration of lost upper-limb function will greatly enhance quality of life. While pharmacologic agents^4^, intense motor retraining^5^, and epidural spinal cord stimulation^6^ have shown promising results for locomotion restoration in the laboratory setting, their potential for upper extremity functional restoration in humans are under active investigation. The efficacy of epidural spinal cord stimulation has been demonstrated in chronic paraplegic SCI subjects, leading to the recovery of both lower-limb volitional control and postural function^7,8^, and has only recently been shown to restore volitional upper extremity function in chronic tetraplegic SCI subjects^9^. Similar results have been attained non-invasively with transcutaneous electrical spinal cord stimulation in paraplegic human subjects^10^. It remains to be determined if combinatory strategies, such as the pairing of electrical stimulation with pharmacological agents^11,12^, will lead to a synergistic therapeutic effect for upper extremity functional restoration as seen in animal models. In this study we examined the effects of stimulation alone or in combination with buspirone, a monoaminergic agonist, in six chronic AIS B cervical spinal cord injured human subjects. Voluntary upper limb function was evaluated through measures of controlled hand contraction, handgrip force production, dexterity measures, and validated clinical assessment batteries.

## Methods

### Enrollment

**Figure 1:**
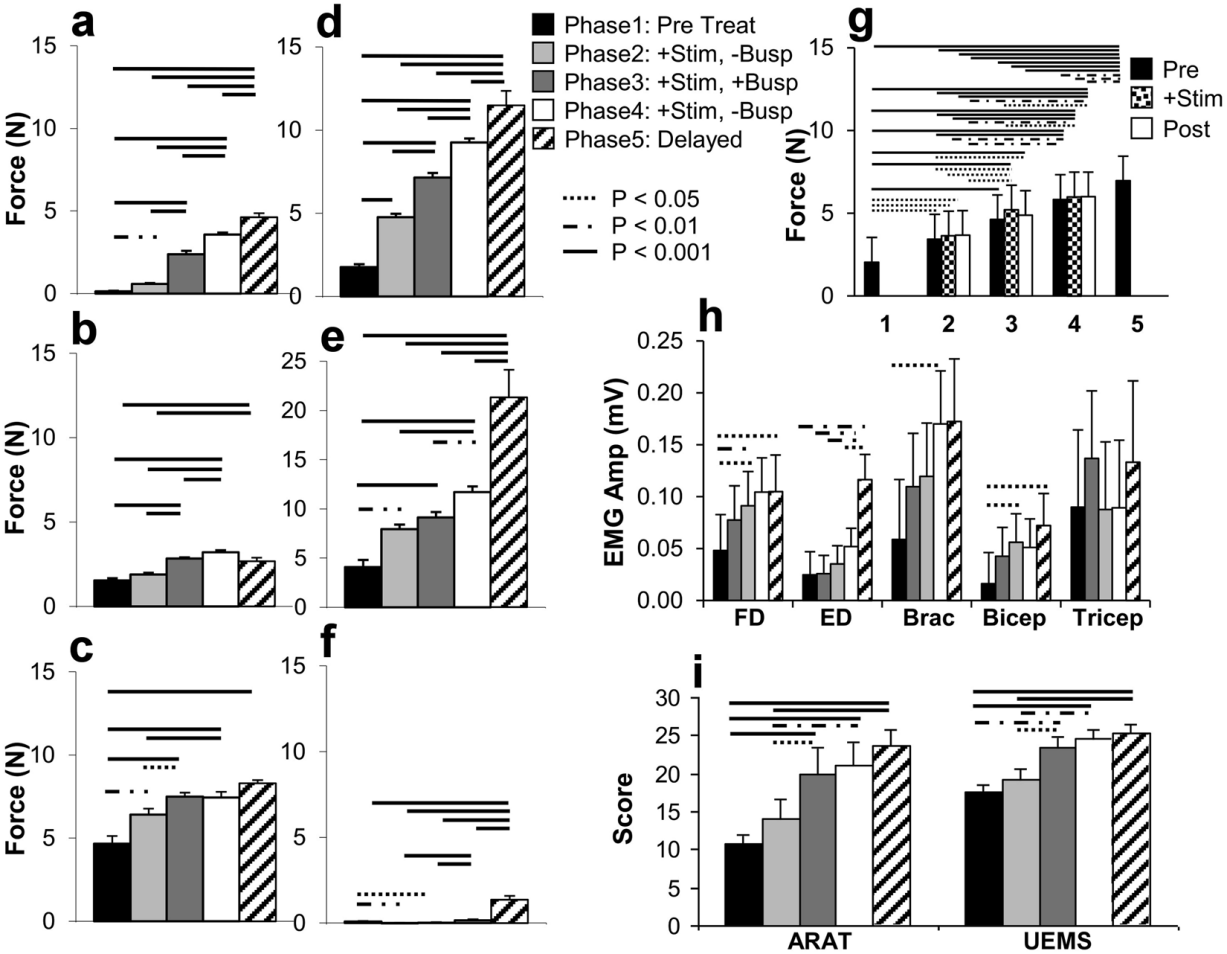
Improvement in hand function with interventions. Assessments of grip strength were made in 6 paralyzed subjects over 6 weeks Pre-Treatment (Phase 1, Pre Treat), followed by testing over 2 weeks with stimulation (Phase 2, +Stim, −Busp), followed by testing over 2 weeks with stimulation plus oral buspirone (Phase 3, +Stim, +Busp), followed by testing over 2 weeks with stimulation plus placebo (Phase 4, +Stim, −Busp), and followed by at least 4 days of testing over 2 weeks Post-Treatment after a 3–6 month delay (Phase 5, Post Treat) (**a**–**f**) (also see Fig. 4e for the number of test sessions and the number of contractions in each Phase). Mean (±SEM) grip strength for all 6 subjects before (Pre), with stimulation (+Stim), and after stimulation (Post) for each phase (**g**), EMG amplitudes as percent increase over baseline (Pre Treat) levels (**h**), ARAT and UEMS (**i**) are shown. (**a**–**f**) show plots for each individual with pre, during, and post averaged within a period. G represents averages of the group means within each phase further divided by baseline (Pre), stimulation (+Stim), and post-stimulation (Post) conditions. Horizontal lines indicate significant differences at *P* < 0.05 (dotted), *P* < 0.01 (dash-dot), or *P* < 0.001 (solid line). FD, flexor digitorum; ED, extensor digitorum; Brac, brachioradialis; Bicep, biceps brachii; Tricep, triceps brachii.

Written informed consent was obtained from each subject prior to enrollment in the study. The University of California, Los Angeles Institutional Review Board approved all procedures associated with this study. All experimental procedures were carried out in accordance with institutional guidelines and regulations. The clinical trial was registered with clinicaltrials.gov prior to any subject enrollment (identifier NCT02313194, registered 09/12/2014). Subjects were enrolled based on the inclusion-exclusion criteria (Table 1) of traumatic cervical spinal cord injury, American Spinal Injury Association Impairment Scale (AIS) B^13^, greater than 12 months from injury, lesion level at C5 or above, and stable motor function as documented by sequential clinical exams (Fig. 1 phase 1). Baseline AIS, upper extremity motor exam portion (UEMS), and Action Research Arm Test (ARAT)^14^ clinical scores were assessed prior to study interventions (Fig. 1i, see also Fig. 4b,c,f). All study subjects were maximized on their pre-existing rehabilitation regimens prior to initiation of baseline testing.

**Table 1:** All research participants, irrespective of age or sex, will meet the following criteria.

Inclusion Criteria	Exclusion Criteria
1. 18 years of age or older. 2. Non-progressive SCI at or above C5 functional level. 3. Be at least one-year post injury. 4. Be unable to grip or move independently, requiring full assistance with all rehabilitation activities and activities of daily living. 5. Segmental reflexes remain functional below the lesion (screening for preservation of lower motoneuron innervation) 6. Female subjects of child-bearing potential must not be pregnant and must be using a medically acceptable method of contraception.	1. Cardiopulmonary disease or dysautonomia that would contraindicate hand/arm movement. 2. Recipients of Botox injections in the prior six months. 3. Disorders or conditions that would require MRI monitoring. 4. Coagulopathy, cardiac risk factors, or other significant medical risk factors for surgery. 5. Prior implantations of neurostimulators, cardiac pacemakers, defibrillators, shunts, stents, or aneurysm clips. 6. Involved in another clinical trial. 7. Ongoing treatments with an anti-spasticity medication regimen. 8. Clinically significant depression or ongoing drug abuse. 9. Painful musculoskeletal dysfunction, unhealed fracture, contracture, pressure sore, or infection that might interfere with upper extremity training.

### Study Design

Subjects were evaluated in five successive study phases beginning with a pre-intervention assessment period of 6 weeks (Phase 1) during which subjects received a placebo. This was followed by evaluation over three consecutive 2-week treatment phases (Phases 2–4). In Phase 2, subjects received stimulation and a placebo whereas in Phase 3, buspirone treatment was combined with stimulation. In Phase 4, subjects continued to receive stimulation, however buspirone treatment was withdrawn and replaced with a placebo. Each subject underwent a post-treatment testing period of 2 weeks (Phase 5) approximately three months after all treatments were discontinued^15^. All subjects and experimenters were blinded to stimulation parameters and pharmacologic therapy in a double-blind manner. All subjects continued to participate in their pre-existing rehabilitation regimens throughout the duration of the study.

### Treatments

Subjects were informed, prior to beginning the study, that they would receive oral buspirone treatments or a placebo. Buspirone was selected based on a minimal side effect profile and its 5HT1A agonist property, an important and prevalent neurotransmitter system for locomotor function^11,16,17^. A direct agonist was chosen instead of a selective serotonin reuptake inhibitor (SSRI) due to the loss of serotonin synaptic terminals in injury that would theoretically render the action of an SSRI ineffective^18^. All subjects were given buspirone (7.5 mg twice daily for 2 weeks beginning the day before biweekly testing) during Phase 3 and a placebo during Phases 1, 2 and 4.

The transcutaneous stimulation device used for stimulation is non-invasive and painless^19^. Stimulation was achieved with non-invasive cutaneous cathode electrodes attached to the dorsal aspect of the neck overlying the C5 vertebra and grounding electrodes placed over the anterior superior iliac spine. Stimulation parameters ranged from 5–30 Hz and 20–100 mA. Varying combinations of these stimulation parameters were systematically assessed to obtain optimum facilitation of voluntary hand contraction by identification of the relative activation levels of the motor pools studied. During each of the three treatment periods (Phases 2–4) a series of nine 3.5-sec maximum handgrip strength tests were performed per treatment session. In each session of Phase 2–4, three contractions were performed without stimulation, followed by three in the presence of stimulation (twice weekly at 30 Hz and 20–40 mA), followed by three without stimulation. The duration of stimulation during each testing session was approximately 15–30 min. The total number of maximum hand contractions was 9–36 and each session lasted 1 to 2 hours. During stimulation the subjects reported a non-painful, tingling sensation down the arms at the higher stimulation intensities at the site of stimulation with some associated tonic paraspinal muscle contractions at the neck.

### Motor Testing

Voluntary motor control performance was assessed using a handgrip force measurement device^20,21^. EMG activity was collected via surface electrodes placed unilaterally on upper extremity muscles including the flexor digitorum, extensor ‘;’ digitorum, brachioradialis, biceps brachii, and triceps brachii. Stimulation and data collection was carried out using the Konigsberg EMG system (Konigsberg, Pasadena, California). Functional assessments by two validated clinical tools, the upper extremity motor assessment portion of the AIS and the ARAT, were performed weekly during each study phase. Upper extremity spasm was assessed by the Modified Ashworth Scale^22^ before and after each testing session. Two experienced examiners who were blind to the treatment phase conducted the scoring.

### Statistics and Data Analysis

Outcome measures (grip strength/hand force, EMG amplitude, ARAT score, and UEMS score) were averaged across all observations for each subject in a given study phase. Group mean changes from baseline for each outcome measure and each subject were compared over the five phases using a repeated measure analysis of variance (ANOVA) model. The Tukey HSD criterion was used for post-hoc comparisons under this model. Quantile-quantile plots of the residual errors confirmed the normal distribution of the data. A two-sided *P* value of *P* < 0.05 was considered significant. Values are reported as means ± standard error of the mean (SEM). Regression analysis and piecewise linear regression was carried out for each subject at each phase to compute the rate of change of grip strength per number of test sessions (per contraction) within a phase. The datasets generated and analyzed during the current study will be available from the corresponding author upon reasonable request at the conclusion of the study.

### Systematic Review

A PubMed search was conducted on existing peer-reviewed literature regarding the use of serotonin agonists, electrical stimulation, or both to effect functional improvement of the upper extremities in human subjects with cervical spinal cord injury. Search terms included “tetraplegia upper limb” in combination with each of “buspirone”, “electrical stimulation,” “serotonin agonist,” and “electrical stimulation serotonin agonist”. We found no reports of studies using buspirone or any other serotonin agonist as a treatment for tetraplegia. There were four studies on the use of brain-computer interfaces as a form of assistive technology for tetraplegics, and 80 papers detailing the positive effects of functional electrical stimulation or neuromuscular electrical stimulation, in which the stimulation is directly applied to the upper limb muscles. In addition, two published studies reported the efficacy of electrical stimulation of the median nerve. One study described a transcutaneous or implanted electrical stimulator applied directly to the spine/spinal cord in an attempt to increase upper extremity function^23^.

### Role of the funding source

The funding sponsors of this study were not involved in the conceptualization of the study protocol, methodology, data analysis, data interpretation, or publication decisions. The corresponding author designed and supervised this study, had full access to all data collected and analysis results, and was responsible for the decision to publish this article.

## Results

### Pre-intervention

**Figure 2:**
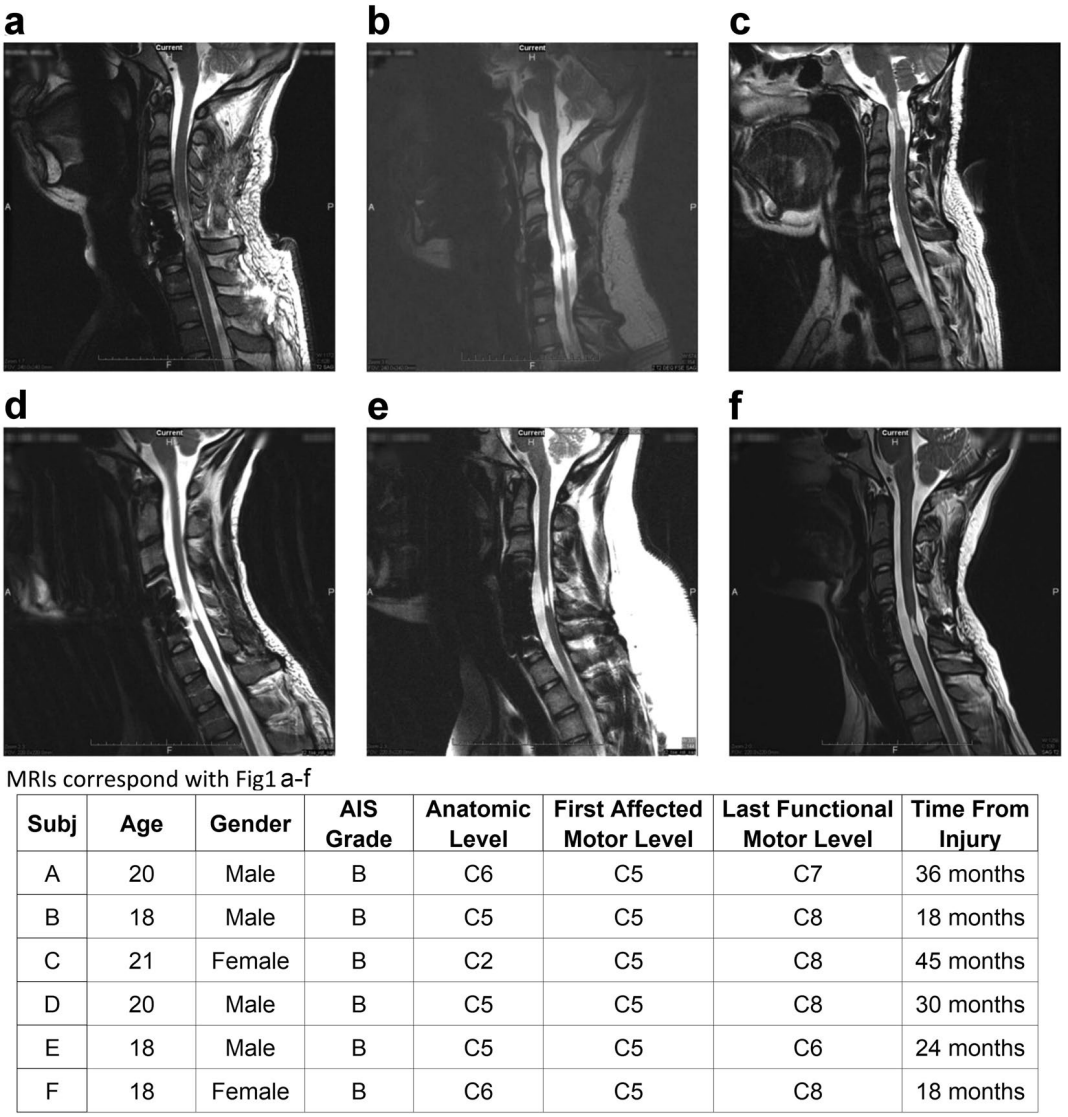
Patient demographic and functional data and sagittal T2 MRI Imaging demonstrating the location of cervical spinal cord injury of subjects (**a**–**f**) at approximately the C2 (**c**), C5 (**b**,**d**,**e**) or C6 (**a**,**f**) spinal levels. The injury location displays a high intensity T2 signal corresponding to a glial scar formation. Spinal cord tissue distal and proximal to the injury locus has a normal appearance without evidence of post-traumatic syrinx formation. The extent of cord atrophy ranges from 62% loss of cord diameter (**c**) to 26% loss of cord diameter (**b**) when compared to adjacent uninjured levels. The glial scar spans approximately one spinal cord level in all subjects with the exception of subject a where the glial scar spans two vertebral levels.

**Figure 3:**
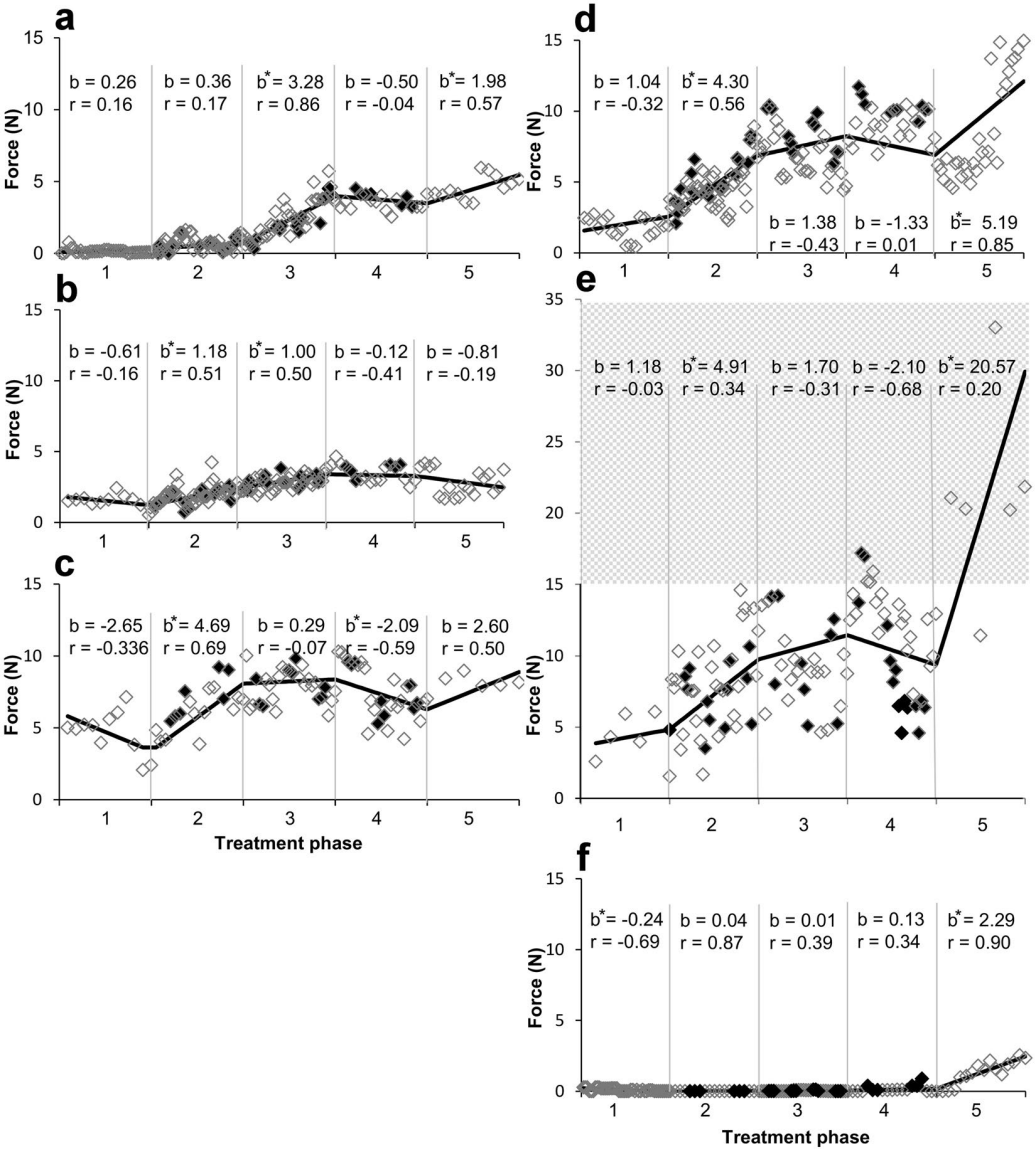
Plot of maximal hand force across contractions during each of the five phases of testing for all six subjects. The slopes in Phase 1 are relatively neutral for all subjects signifying a lack of improvement in force during Phase 1. There is a total of at least 10 sessions (90 hand contractions) with data recorded during at least 3 sessions at the beginning, middle, and end of Phase 1. In general, there were more sessions in Phase 2 due to the fine-tuning of the simulation parameters for each subject. Piecewise linear regression lines are drawn within each phase. Solid diamonds represent stimulation on, unfilled diamonds represent stimulation off; b = slope; r = correlation coefficient; * denotes p < 0.01 for slope (rate) compared to zero. N = Newton, X-axis numerical values represent the phases of the study. Shaded area in e highlights the forces above 15 N.

**Figure 4:**
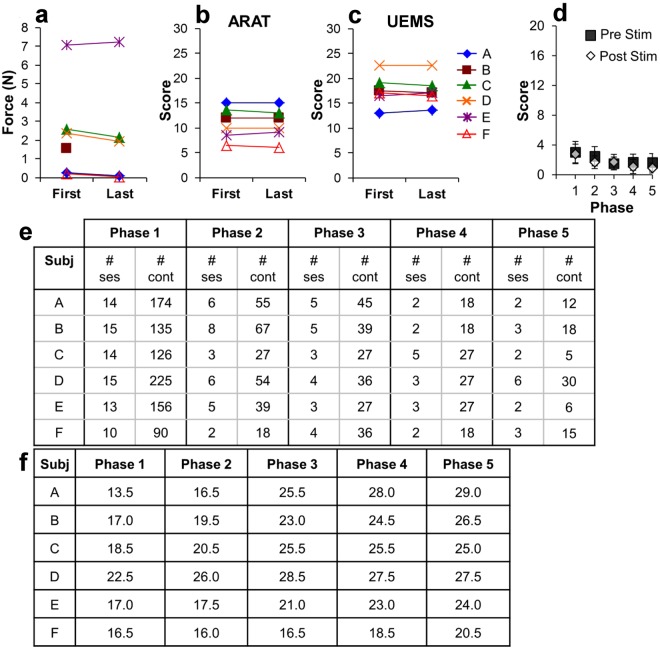
Baseline testing (Phase 1) demonstrates stable hand function by handgrip device (**a**), ARAT (**b**), and UEMS (**c**) scores prior to initiation of any intervention. Upper extremity modified Ashworth scores were assessed during each session before (squares) and after (diamonds) testing (**d**). The total number of sessions and hand contractions and the specific UEMS are shown in (**e**,**f**), respectively. Maximal handgrip force on the last day of testing was not different from the first day of testing in the 6 subjects (**a**). Testing during this phase spanned a period of 6 weeks with bi-weekly testing sessions. Each data point represents an average of 3 maximal handgrip contractions. Clinical testing by ARAT (**b**) and UEMS (**c**) scores demonstrate no evidence of improvement during this initial baseline-testing period. There was a general decrease in upper extremity spasms within each testing session and as the study progressed (**d**). During baseline Phase 1 testing, there were more sessions and hand contractions than for all of Phases 2–4 combined (**e**). Note that the variability in the number of sessions and contractions among subjects and across different Phases was inevitable as a result of complications of co-morbidities associated with SCI, i.e., urinary tract infections, pressure ulcers, etc. During Phase 1, EMG was not recorded during all sessions due to the relative stability of the response: consequently, there are a higher number of contractions during Phase 1 compared to that shown in Fig. 3.

All six subjects demonstrated minimal upper extremity function at baseline (Fig. 1a–g) with average maximum grip strength ranging from 0.7 N (2% normal) (Fig. 1f) to 4.7 N (16% normal) (Fig. 1c) when normalized for patient age and gender^24^. Subjects, at least 18 months from injury, have MRI confirmed, non-progressive, motor-complete injury lesion (Fig. 2). Baseline maximum grip strength, ARAT and UEMS scores did not demonstrate any improvement during Phase 1 bi-weekly testing (Figs 3 and 4a–c) despite completing at least ten baseline testing sessions with a minimum of nine measured contractions per session (Phase 1, Figs 3 and 4e). Initial UEMS varied from 13.5 to 22.5 averaging 17.5 (Fig. 4c,f) and did not correspond with the degree of cord atrophy, extent of glial scar, and level of injury according to MR imaging (Fig. 2). Initial ARAT scores ranged from 6.1 to 15, averaging 10.8 (Fig. 4b).

### Intervention

During Phase 2, stimulation alone at the C5 spinal segment increased hand strength in five subjects compared to baseline (Fig. 1a–g). Four of the five subjects displayed statistically significant improvement in maximum hand grip force, over the course of Phase 2 (Fig. 3b–e). Despite this finding, EMG amplitude of individual muscle groups did not show a significant difference when compared to baseline measurements in Phase 1 (Fig. 1h), although average amplitudes were increased in all tested muscle groups. Phase 2 UEMS were improved for five of six subjects (Fig. 4f) but were not significantly increased in aggregate (Fig. 1i).

When buspirone treatment was added to the established stimulation regimen in Phase 3, four subjects demonstrated increased hand strength compared to Phase 2 and five subjects demonstrated improvement compared to Phase 1 (Fig. 1a–e). The remaining subject demonstrated significantly decreased hand strength compared to Phase 1 baseline (Fig. 1f). Two subjects demonstrated significant progressive improvement in grip strength over the course of the treatment phase (Fig. 3a,b). Flexor digitorum and biceps EMG amplitudes were significantly improved when compared to baseline measurements (Fig. 1h). Phase 3 UEMS were improved for all subjects (Fig. 4f) and were significantly improved in aggregate as compared to baseline and Phase 2 assessments. ARAT scores were also improved in aggregate compared to baseline and Phase 2 assessments (Fig. 1i).

**Figure 5:**
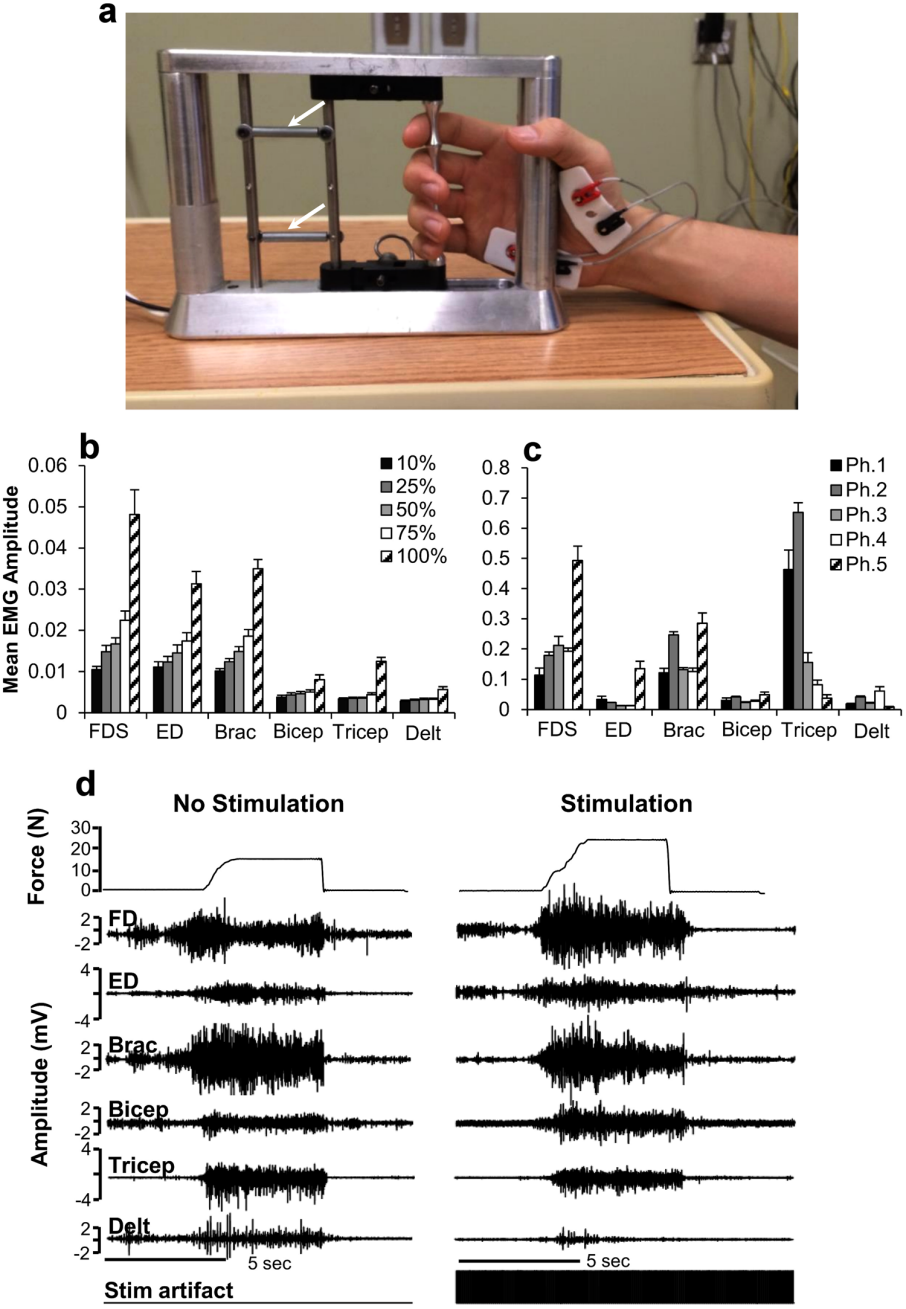
EMG profiles during hand contractions in a normal subject and Subject D. Representative photo of the handgrip device (**a**). Different spring(s) (arrows) were used to adjust the resistance level of the handgrip. The normal subject was asked to contract his hand in response to perceived percent effort (10, 25, 50, 75, 100%) and normal EMG responses are shown (**b**). The average EMG throughout the sessions during Phase 1–5 were shown for Subject D (**c**). Raw EMG tracings of Subject D during a maximal hand contraction in Phase 3 with and without stimulation (**d**) are shown. In normal subjects, there is a predominant activation of distal arm muscles (FD, ED, Brac) and a lesser activation of proximal arm muscles (Bicep, Tricep, and Delt) during a hand contraction (**b**). There also is an increase in EMG amplitude as the percentage of effort increases from 10% to 100%. In Subject D, there is a predominant activation of the Tricep during a hand contraction in the early phases of testing, particularly in Phases 1 and 2 (**c**). During the later phases, particularly Phase 5, there is an increase in the activation of FD, ED, and Brac during hand contraction (**c**), closely mimicking the pattern observed in the normal subject (**b**). The handgrip force and EMG during a maximum handgrip performed with (11 N) and without (7 N) stimulation show increased activation in the FD and ED and relative relaxation of the Tricep and Delt to impart a greater force (**d**). There is no evidence of spastic activity as the hand contraction is performed. FD, flexor digitorum; ED, extensor digitorum; Brac, brachioradialis; Bicep, biceps brachii; Tricep, triceps brachii; Delt, deltoid. Ph, Phase.

In Phase 4, stimulation following the withdrawal of buspirone resulted in an increase in strength in five subjects compared to Phase 3. In five subjects, grip strength was also significantly greater in Phase 4 than in Phase 1 (Fig. 1a–f). For all subjects combined, the mean grip strength consistently displayed an upward trend with each successive treatment phase (Fig. 1g). However, the relative response to each intervention varied among subjects (Fig. 3). On average, there was no significant difference in grip strength with stimulation on or off for each treatment phase (Fig. 1g). Digit flexor and extensor EMG amplitudes also increased progressively across phases along with grip force (Fig. 1h, see also Fig. 5c,d). The increase in hand force occurred without EMG evidence of spasm-induced contractions (Fig. 5d). Overall, EMG activation patterns corresponding well with that of a normal subject (Fig. 5b,c). The aggregate Modified Ashworth score, reflecting upper extremity spasticity, was reduced with each study phase throughout the course of the study (Fig. 4d).

### Post-intervention

During Phase 5, after cessation of all treatment for 3 to 6 months, all subjects demonstrated significant and persistent improvement in hand grip force as compared to baseline; while improvements compared to all other treatment phases were generally present but more variable (Fig. 1a–g). Average maximum grip strength in Phase 5 ranged from 1.3 N (4% normal, Fig. 1f) to 21.4N (46% normal, Fig. 1e). In three subjects, continued significant improvement in grip strength was observed in Phase 5 despite cessation of all treatments (Fig. 3). Average flexor digitorum, extensor digitorum, and biceps EMG amplitudes during Phase 5 were significantly increased from baseline for all subjects (Fig. 1h). Aggregate (Fig. 1i) and individual (Fig. 4f) Phase 5 UEMS and ARAT scores were also significantly increased compared to baseline.

## Discussion

Although stimulation and pharmacological agents have been successfully applied to lumbar spinal circuits to enable locomotor performance in the laboratory and clinical settings^10,11,25^, it was unclear if such approaches are translatable to the cervical spinal circuits governing motor functions in the upper limbs. This study demonstrates a significant level of functional improvement via a combined approach. While the present study does not definitively prove the independent effect of each intervention, it suggests that these interventions are highly interactive and perhaps synergistic as observed in animal experiments^11,12,26–28^. The improvements are unlikely to be due to natural recovery or from repeated practice alone as large cohort studies of SCI patients have demonstrated that the majority of functional improvements occur within 6 months of the injury and that minimal recovery of function is observed past the 12 month time-point^29,30^. All of our subjects were at least 18 months beyond the initial injury and baseline motor function was stable for a 6 week testing period prior to any therapeutic intervention (Phase 1, Fig. 3), with the number of hand contractions greater than all the subsequent treatment phases (Phases 2–4) combined (Fig. 4e). Furthermore, our previous study demonstrates stability of function after handgrip rehabilitation^31^. Concomitant to significantly improved hand strength, gains in upper extremity functional metrics by ARAT (13 point improvement, minimum clinically significant difference: 6 points^32^) and UEMS (7 point improvement, minimum detectable change: 2.72 points^33^) tests reflect the impact of these interventions on the patient’s overall upper extremity motor function (Fig. 1i). Functional gains demonstrated by subjects during intervention (Fig. 1, Phases 2–4) appear to be durable and persist even after withdrawal of stimulation (Phase 4) and all treatment interventions (Phase 5). This suggests that the novel interventions reengaged previously unavailable motor pools. With training, these motor pools become functionally active and integrated daily skills. The persistence of improved hand function following buspirone withdrawal (Phase 3) is consistent with our previous finding that in cats trained to step after spinal cord transection, stepping ability persists for 6 weeks without further training^34^.

While the precise mechanism remains to be determined and is beyond the scope of this study, our working hypothesis is that stimulation and buspirone interventions enhance the level of excitability of pre-motor spinal circuitries that mediate hand function. Cervical spinal stimulation as used here largely affects interneuronal pathways that generate action potentials among motoneurons within a motor pool in a more normal stochastic time frame^35^. As such, stimulation is hypothesized to potentiate the generation of miniature excitatory potentials and thus shifts the spinal motor network excitability closer to a motor threshold. This results in a wide population of interneurons which are asynchronously activated in a randomized pattern relative to each stimulation pulse. Therefore, the more optimal stimulation parameters seem to be those where stimulation “enables” the supraspinal-proprioceptive input to generate a highly non-synchronous activation among the motoneurons within a motor pool^7^.

The present results suggest that non-functional networks in the chronically injured cervical cord can become re-engaged and progressively improve motor performance. These findings are significant from the following perspectives: 1) the cervical spinal circuitry can be neuromodulated to enhance maximum neuromuscular force as well as fine control of movements in the upper limb after paralysis; 2) the improvements in multiple functional measures were associated with an enhancement in the performance of daily upper extremity tasks (ARAT); 3) even after more than a year of inactivity of the sensorimotor circuits, significant levels of activity-dependent plasticity persisted; and 4) these observations raise the possibility that the neuromodulatory concept could apply to other neural networks and therefore could be applied to neuromotor disorders such as stroke and Parkinson’s disease.

While this study is not directly comparable to existing investigations of epidural spinal cord stimulation, it highlights notable differences between the two modalities. Most salient is the capability to effect durable improvement in upper extremity neuromuscular force and control through non-invasive means. Similar outcomes have been attained with epidural stimulation^9^, but such functional improvements required an invasive intervention with all associated costs and risks. Transcutaneous techniques also afford the possibility to adjust targeting over a wider range of cervical levels whereas epidural stimulation is limited by the extent of the pre-implanted stimulator. Conversely, epidural stimulation is not associated with significant pain or paresthesia, permits better spatial resolution, and is not inhibited in patients with a large body habitus. A study directly comparing these two modalities will be essential to determine clinical superiority.

A recent investigation has described the use of non-invasive transcutaneous stimulation for upper extremity functional restoration in cervical SCI^23^. Despite treating a patient population with a higher injury grade, we have observed a similar extent of functional improvement to the preceding study. These findings suggest that transcutaneous spinal cord stimulation is broadly applicable in the SCI patient population and reinforce the concept that functional connections persist even in the most severe injuries. While further investigation will be necessary in subjects with more varied injuries, it is possible that the novel pharmaceutical treatment introduced in our study is the reason for the parity in outcomes.

## Conclusions

This study demonstrates that upper extremity function in chronic SCI human subjects with tetraplegia can be improved through neuromodulation of the cervical cord with pharmacological and/or spinal electrical stimulation. We conclude that the serotonin agonist Buspirone and electrical stimulation of the cervical cord can improve the strength and voluntary control of the upper limbs in chronic tetraplegic subjects, resulting in extended functional gains. Our subjects experienced minimal to no side effects from the treatment drug. Although similar methods have been used to improve locomotion and posture in paraplegic subjects, these results confirm that the cervical spinal circuits can similarly be reengaged through electrical and/or pharmacological stimulation.
